# Foxtail Millet [*Setaria italica* (L.) Beauv.] Grown under Low Nitrogen Shows a Smaller Root System, Enhanced Biomass Accumulation, and Nitrate Transporter Expression

**DOI:** 10.3389/fpls.2018.00205

**Published:** 2018-02-22

**Authors:** Faisal Nadeem, Zeeshan Ahmad, Ruifeng Wang, Jienan Han, Qi Shen, Feiran Chang, Xianmin Diao, Fusuo Zhang, Xuexian Li

**Affiliations:** ^1^Key Laboratory of Plant–Soil Interactions, Ministry of Education, Department of Plant Nutrition, China Agricultural University, Beijing, China; ^2^Institute of Crop Sciences, Chinese Academy of Agricultural Sciences, Beijing, China

**Keywords:** foxtail millet (FM), low nitrogen (LN), root architecture, nitrogen uptake, nitrogen transport

## Abstract

Foxtail millet (FM) [*Setaria italica* (L.) Beauv.] is a grain and forage crop well adapted to nutrient-poor soils. To date little is known how FM adapts to low nitrogen (LN) at the morphological, physiological, and molecular levels. Using the FM variety Yugu1, we found that LN led to lower chlorophyll contents and N concentrations, and higher root/shoot and C/N ratios and N utilization efficiencies under hydroponic culture. Importantly, enhanced biomass accumulation in the root under LN was in contrast to a smaller root system, as indicated by significant decreases in total root length; crown root number and length; and lateral root number, length, and density. Enhanced carbon allocation toward the root was rather for significant increases in average diameter of the LN root, potentially favorable for wider xylem vessels or other anatomical alterations facilitating nutrient transport. Lower levels of IAA and CKs were consistent with a smaller root system and higher levels of GA may promote root thickening under LN. Further, up-regulation of SiNRT1.1, SiNRT2.1, and SiNAR2.1 expression and nitrate influx in the root and that of SiNRT1.11 and SiNRT1.12 expression in the shoot probably favored nitrate uptake and remobilization as a whole. Lastly, more soluble proteins accumulated in the N-deficient root likely as a result of increases of N utilization efficiencies. Such “excessive” protein-N was possibly available for shoot delivery. Thus, FM may preferentially transport carbon toward the root facilitating root thickening/nutrient transport and allocate N toward the shoot maximizing photosynthesis/carbon fixation as a primary adaptive strategy to N limitation.

## Introduction

Nitrogen (N) is an essential macronutrient for plant growth, development, and production. As an essential component of nucleic acids and proteins, N actively participates in most physiological and biological processes in crop production including photosynthesis, carbohydrate allocation, root patterning, and flower development, and hence signifies itself as a critical macronutrient controlling crop yield and quality (Stitt, 1999; Miller and Cramer, 2004). In agricultural systems the natural availability of soil N often limits crop yield owing to the fact that most non-legume plants need to absorb 20–50 g of N by their roots to produce 1 kg of dry biomass (Robertson and Vitousek, 2009). It is estimated that almost 10^11^ kg of N per annum is applied as fertilizers in agricultural systems globally (Glass, 2003). N concentrations in the soil solution, as nitrate and ammonium, range from 100 μM to 10 mM. This heterogeneity and dynamic variations of N concentrations lead plants to sense external N availability and respond accordingly via hierarchical morphological, physiological, and molecular adaptations (Glass, 2003; Miller et al., 2007; Garnett et al., 2009). Legumes enhance N uptake through nodulation and N fixation (Postgate, 1998), while many non-legume crops, i.e., maize, enhance root growth for N forage when grown in local LN environment (Wang et al., 2003; Chun et al., 2005a). However, different adaptive strategies may have arisen in different crop species over evolution.

Foxtail millet (FM) [*Setaria italica* (L.) Beauv.], one of the world’s oldest crop, was domesticated in China approximately 8700 years ago. It was named FM due to bushy, tail-like appearance of its immature panicles. Throughout areas in southern Europe and Asia, it provides 6 million tons of grain for people and ranks second in total world millet production (Li and Wu, 1996; Yang et al., 2012). In dry regions of north China, it is one of the main food crops (Wang C. et al., 2012). FM can be grown in semi-arid regions (Hancock Seed Co, 2014). Owing to its high tolerance to drought and low fertility, FM is being studied as a model crop for cereals (Veeranagamallaiah et al., 2008; Doust et al., 2009; Diao et al., 2014). Unlike other C4 grasses, FM has a small genome size (∼490 Mbp), small plant size, and a quick generation time which values it as a model system. As a consequence, genotypes Yugu1 and Zhang gu have been used to compile two full-reference genome sequences (Bennetzen et al., 2012; Zhang et al., 2012).

Acclimation responses of FM to LN at the seedling stage, concerning root architectural traits, have not been previously reported. Furthermore, literature regarding other morphological and physiological acclimation responses of FM to LN is unavailable. The objective of this study was to analyze morphological and physiological responses of FM to short-term N limitation, providing selective traits for N-efficient FM breeding. Interestingly, we found that FM is an extremely large-root crop and responds to LN at least by enhancing expression of related nitrate transporters.

## Materials and Methods

The experiment was conducted in a standard growth chamber at China Agricultural University, Beijing, China. Seeds of a standard FM variety Yugu1 (Cheng and Dong, 2010) were first washed with deionized water three times. Second, these seeds were sterilized with 10% H_2_O_2_ for 30 min and third, imbibed in saturated CaSO_4_ solution with aeration for 5 h. After that the seeds were germinated on moist filter paper in the growth chamber. The seedlings with the 2-cm primary root were wrapped using filter paper, enfolded into a two-layer cylinder saturated with deionized water, placed vertically in a growth holder containing deionized water, to ensure continuous supply of deionized water to the seedlings, and covered with black plastic until leaf emergence. Consistent and uniform seedlings having two fully expanded leaves were transferred into continuously aerated 3.4-L container with nutrient solution strength of 25% from days 1 to 3, 50% from days 4 to 7, and 100% from days 8 to 14. Seedlings were subjected to LN from days 15 to 21. There were four seedlings transferred per pot. The whole nutrient solution (as CK) consisted of: 2 mM NH_4_NO_3_, 0.25 mM KH_2_PO_4_, 0.75 mM K_2_SO_4_, 0.1 mM KCl, 2 mM CaCl_2_, 0.65 mM MgSO_4_, 0.2 mM Fe-EDTA, 1 × 10^-3^ mM MnSO_4_, 1 × 10^-3^ mM ZnSO_4_, 1 × 10^-4^ mM CuSO_4_, 5 × 10^-6^ mM (NH_4_)_6_Mo_7_O_24_, and 1 × 10^-3^ mM H_3_BO_3_. To make the N limitation medium (as LN), NH_4_NO_3_ was reduced to 0.02 mM in the nutrient solution, and other nutrients remained unchanged in concentration. The pH was maintained at 6.0. Each treatment had six biological replicates and the nutrient solution was changed every 2 days. The experiment was reproduced three times.

The conditions in standard growth chamber were as follows: temperature was 28/22°C, relative humidity was 65%, illumination was 300 μmol photons m^-2^ s^-1^ and photoperiod was 14/10 h (day/night). Root and shoot samples were harvested on 21st day after transfer to the nutrient solution. For RNA isolation, soluble proteins, free amino acids and total soluble sugars, and shoot samples were harvested, frozen immediately in liquid N_2_, and stored at -80°C while root samples were carefully washed three times with deionized water, wiped gently using a blot paper prior to immediate freezing in liquid N_2_, and storage at -80°C. Samples were harvested and washed three times, oven-dried at 105°C for 30 min, then dried at 70°C until constant weight for dry weight (DW) analysis and other physiological measurements.

### Soil and Plant Analyzer Development (SPAD) Values of Leaves

On the day of harvest, SPAD values were measured at 8:00–9:00 a.m. using a Chlorophyll Meter (SPAD-502, Konica Minolta Sensing Inc., Japan). The youngest fully expanded leaf (the 4th leaf) was analyzed three times with the SPAD meter. The process was carried out for all four plants in every pot. The average of three SPAD reads from the same leaf was taken as the SPAD value for that leaf. Four averages from every pot were considered as technical replicates. Each treatment had six biological replicates (six pots).

### Root Architecture

The whole root was divided into crown roots and lateral roots. Each kind of roots was counted manually and measured with a ruler. A scanner (Epson 1680, Indonesia) was used to scan the roots and the scanned images were analyzed using the WinRHIZO software (version 5.0) (Regent Instruments Inc., Quebec City, QC, Canada) to get total root length and average diameter following previously described methods (Peng et al., 2010). Lateral root density was defined as the number of lateral roots per unit length of crown root with lateral roots. Each treatment had six biological replicates.

### Analysis of the N Concentration in the Shoot and Root

The dry samples of shoot and root were weighed and ground into fine powder. The ground sample of 0.3 g was digested by H_2_SO_4_–H_2_O_2_ followed by total N analysis using a modified Kjeldahl digestion method (Baker and Thompson, 1992).

### Measurement of Total Free Amino Acids, Soluble Proteins, Total Soluble Sugars, and C/N Ratio

A standard kit (Coomassie Protein assay reagent; Bio-Rad, Hercules, CA, United States) with bovine serum albumin as a reference was used to extract and analyze soluble proteins. Total free amino acid concentration was measured according to the Rosen ninhydrin colorimetric method using leu as a standard (Rosen, 1957). A commercially available kit (Boehringer Mannheim, Germany) was used to determine the concentration of soluble sugars. The C/N ratio was analyzed by loading ∼50 mg finely ground shoot or root tissue into Elementar vario Macro CN analyzer (Elementar Technologies, Hanau, Germany).

### Hormone Extraction and Quantification by Enzyme-Linked Immunosorbent Assay (ELISA)

Approximately 0.5 g fresh shoot or root powder was homogenized in 2 ml of 80% methanol (containing 40 mg l^-l^ butylated hydroxytoluene as an antioxidant), incubated at 4°C for 48 h, and then centrifuged at 1900 × *g* at 4°C for 15 min. The supernatant was passed through C18 Sep-Pak Cartridges (Waters Corp.), and the hormone fraction was eluted with 10 ml of 100% (v/v) methanol and then 10 ml ether. The elute was N_2_-dried at 20°C. The N_2_-dried extracts were dissolved in 2.0 ml phosphate-buffered saline (PBS) containing 0.1% (v/v) Tween-20 and 0.1% (w/v) gelatin (pH 7.5) to analyze the concentration of free IAA, ABA, GA, and CKs by ELISA following a well-established protocol (Weiler et al., 1981).

### Relative Gene Expression

Following manufacturer’s protocol (Invitrogen), Trizol reagent was used to extract total RNA from shoot and root samples of FM. To remove any possible DNA contamination, total RNA (4–5 g) was digested by DNase 1 (Takara Biomedicals, Kyoto, Japan). Reverse transcription of RNA samples into cDNA was carried out using M-MLV reverse transcriptase (Invitrogen). Quantitative real-time PCR (qRT-PCR) in a Bio-Rad iCycler iQ5 system (Bio-Rad, Hercules, CA, United States) was used to determine gene expression levels using the SYBR^®^ Premix Ex TaqTM (Takara) along with specific primers constructed according to the genes of interest having elongation factor 1-α (EF-1α) as internal control or reference gene (**Table 1**). The primers were designed using Primer Premier 5.0. The program was as follows: pre-incubation for 10 min at 95°C, 40 cycles of determination at 95°C for 15 s, annealing at 60°C for 30 s, and finally, extension at 72°C for 30 s. The standard comparative ΔΔ*C*(*t*) method (Livak and Schmittgen, 2001) was used to calculate relative gene expression levels. The treatments used for the analysis had four biological replicates and three technical replicates of every biological replicate. The IDs and names of genes examined were given in **Table 1**.

**Table 1:** Candidate genes and related information for qRT-PCR analysis.

Gene symbol	Description	Gene ID	Primer sequence	Product size (bp)
SiNRT1.1	Nitrate transporter 1.1	Seita.9G327900	F: TGACTTGGTTTTGGCGTGTA R: ACGCTTCCATTCATTCCAAG	200
SiNRT2.1	Nitrate transporter 2.1	Seita.1G115700	F: CCTGCTGTTTGTGTTTGTGC R: TGAACCCTTGTGCACCTACT	187
SiNRT1.11	Phloem transporter 1.11	Seita.3G406400	F: AATCGCCAAGTGCTATGGTC R: CATGACAGCAGAAGCAGAGC	220
SiNRT1.12	Phloem transporter 1.12	Seita.3G243200	F:TCAAGTAGCTTGGTGGTTGCT R: ACTCCTGCATTTCTCGAACA	170
SiNAR2.1	Nitrate assimilation-related 2.1 (NAR2.1/NRT3.1)	Seita.1G218500	F: GACAAGGCGTGCCAGTTC R: CGTAGTAGGTGCCCGAGG	106
SiAMT1.1	Ammonium transporter 1.1	Seita.1G237300	F: GTCCTTCCTCACCATCCT R: CGTTCCAGTGCCCCGTCT	157
SiAMT1.3	Ammonium transporter 1.3	Seita.1G189700	F: CCCAAACTCGAGAGGTGCAT R: TGCACATTCCACATTCCTCCA	194
EF-1α	Elongation factor 1-alpha	Si022039m	F: TGACTGTGCTGTCCTCATCA R: GTTGCAGCAGCAAATCATCT	133

### ^15^N-Labeled ${\mathrm{NH}}_{4}^{+}$ and ${\mathrm{NO}}_{3}^{–}$ Influx Studies

To analyze N uptake by FM seedlings, CK and LN roots were rinsed inside the 1 mM CaSO_4_ solution for 1 min, and then transferred to uptake solution containing 200 μM/2 mM ^15^(NH_4_)_2_SO_4_ (99.12 atom% ^15^N) or 400 μM/4 mM K^15^NO_3_ (99.29 atom% ^15^N) for 10 min. No K^+^ was added in nutrient solution containing 4 mM K^15^NO_3_ while, to compensate K^+^, 0.6 mM K_2_SO_4_ was added in nutrient solution containing 400 μM K^15^NO_3_. Roots were again rinsed with 1 mM CaSO_4_ solution for 1 min before harvest and dried at 70°C for 48 h. ^15^N accumulation in the root was analyzed by isotope ratio mass spectrometry (DELTAplus XP, Thermo Finnigan).

### Statistical Analysis

Data were analyzed using the one-way PROC ANOVA in SAS (Sas Institute Inc., 2004). Means of different treatments were compared using the least significant difference at a 0.05 or 0.01 level of probability.

## Results

### Plant Growth and N Accumulation in the Control and Low Nitrogen (LN) Plants

**FIGURE 1:**
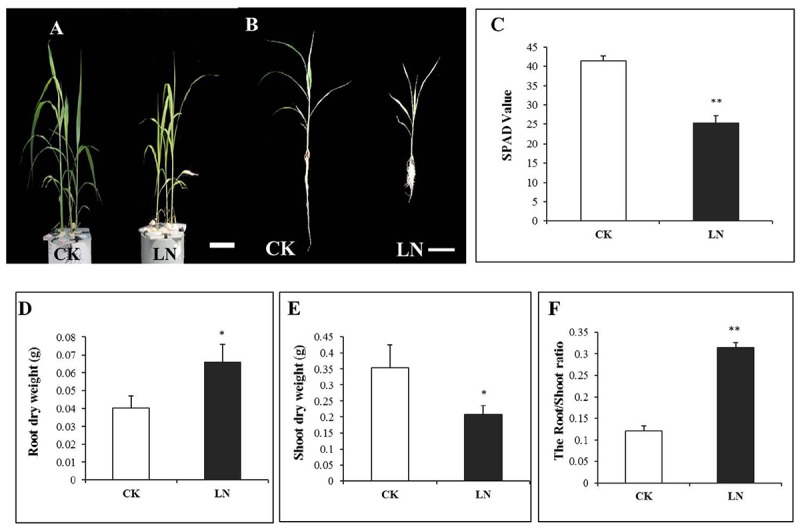
Shoot and root responses of foxtail millet (FM) [*Setaria italica* (L.) Beauv.] to LN. Plants grown under CK and LN in hydroponic pots **(A)**, scale bar at bottom represented a length of 10 cm, plants with the intact root and shoot **(B)**, scale bar at the bottom represents a length of 10 cm, SPAD value **(C)**, root dry weight (DW) **(D)**, shoot DW **(E)**, and root/shoot ratio **(F)**. Error bars represented standard error of six biological replicates (^∗^*P* < 0.05; ^∗∗^*P* < 0.01).

Plants were harvested after 1 week of the LN treatment. LN reduced FM growth (**Figure 1A**) with a particularly shorter root system (**Figure 1B**). Chlorophyll content of leaves is a crucial indicator of internal N nutritional status and is directly correlated to the SPAD value (Sibley et al., 1996; Chapman and Baretto, 1997). SPAD values of the fourth leaf of LN plants were significantly lower compared to those of control plants (**Figure 1C**), indicating reduction of Chlorophyll levels in the LN leaves. In contrast to dramatic reduction in the shoot DW, the root DW nearly doubled under LN (**Figures 1D,E**), resulting in a twofold increase in the root/shoot ratio of LN plants (**Figure 1F**).

**FIGURE 2:**
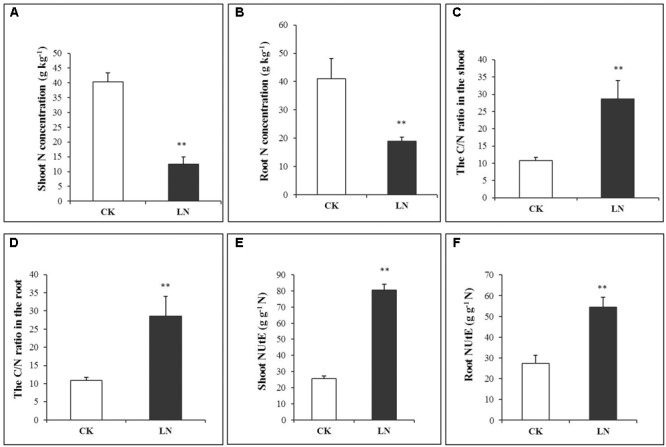
N accumulation in control and LN FM. Shoot N concentration **(A)**, root N concentration **(B)**, C/N ratio in the shoot **(C)**, C/N ratio in the root **(D)**, NUtE in the shoot **(E)**, and NUtE in the root **(F)** in response to LN. Error bars represented standard error of six biological replicates (^∗^*P* < 0.05; ^∗∗^*P* < 0.01).

The N concentration decreased by 69% in the shoot and 54% in the root under LN (**Figures 2A,B** and Supplementary Table 1), indicating more N allocation or remobilization toward the root under LN. The C/N ratio was at the similar level in the shoot and root of the control plants, with a nearly twofold increase under LN (**Figures 2C,D**). Although LN inhibited plant growth and reduced N accumulation (**Figures 1, 2**), the N utilization efficiency, defined as cumulative biomass per unit of N (NUtE, g DW g^-1^ N), increased from 25.51 to 80.65 g g^-1^ N in the shoot and from 27.48 to 54.48 g g^-1^ N in the root under LN, suggesting a higher N utilization efficiency in the shoot (**Figures 2E,F** and Supplementary Table 1).

### Physiological Responses of Foxtail Millet to LN

**FIGURE 3:**
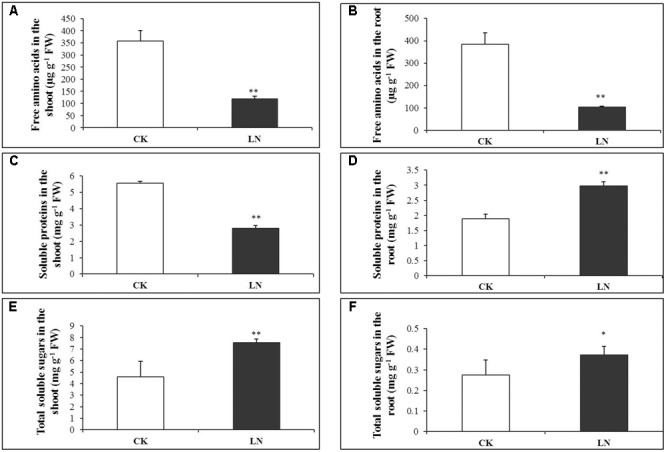
Physiological responses of FM to LN. Soluble proteins in the shoot **(A)**, root **(B)**, free amino acids in the shoot **(C)**, root **(D)**, total soluble sugars in the shoot **(E)** and root **(F)**. Error bars represented standard error of six biological replicates (^∗^*P* < 0.05; ^∗∗^*P* < 0.01).

Low nitrogen had negative effects on accumulation of free amino acids, and the concentration of total free amino acids decreased by 67% in the shoot and 73% in the root under LN (**Figures 3A,B** and Supplementary Table 3). Consistently, the concentration of soluble proteins was reduced by almost 50% in the shoot of the LN plants (**Figure 3C** and Supplementary Table 4), in sharp contrast, it was significantly greater in the root under LN (**Figure 3D**). Carbon metabolism is closely related to N metabolism, and LN caused significant increment in the concentration of total soluble sugars in the shoot and root (**Figures 3E,F** and Supplementary Table 5).

**FIGURE 4:**
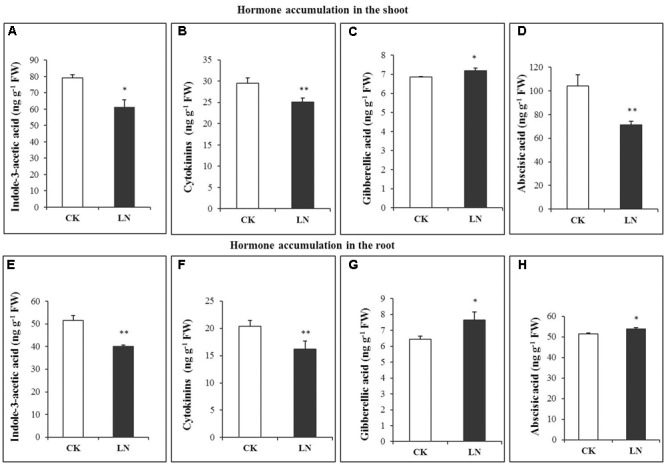
Hormonal responses of FM to LN. Indole-3-acetic acid (IAA) **(A)**, abscisic acid (ABA) **(B)**, gibberellic acid **(C)**, cytokinins **(D)** in the shoot and IAA **(E)**, ABA **(F)**, gibberellic acid **(G)**, and cytokinins **(H)** in the root. Error bars represented standard error of six biological replicates (^∗^*P* < 0.05; ^∗∗^*P* < 0.01).

Hormones are crucial regulators of plant growth and development (Marsch-Martinez and de Folter, 2016). The pattern of accumulation of indole-3-acetic acid (IAA/auxin), cytokinins, gibberellic acid (GA), and abscisic acid (ABA) differed significantly under the LN treatment. LN caused significant decreases in accumulation of IAA in the shoot and root (**Figures 4A,E**). A similar trend was observed in the cytokinin concentration in spite of reduction variations in the shoot and root (**Figures 4B,F**). Contrary to IAA and cytokinin accumulation, the GA concentration was much greater in the shoot and with a slight increase in the root under LN (**Figures 4C,G**). ABA showed a contrasting pattern under LN, with significantly lower (in the shoot) and higher (in the root) concentrations (**Figures 4D,H**).

### Root Architectural and Transport Alterations under LN

**FIGURE 5:**
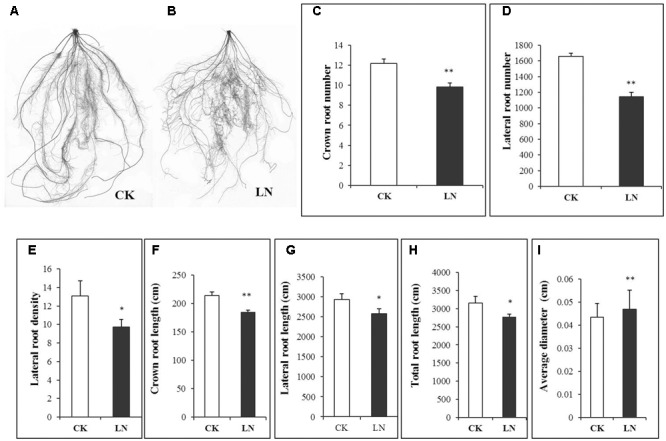
Effects of LN on root morphological characters in FM. Scanned images of the root under CK **(A)** and LN **(B)**, crown root number **(C)**, lateral root number **(D)**, lateral root density **(E)**, crown root length **(F)**, lateral root length **(G)**, total root length **(H)** and average diameter **(I)**. Error bars represented standard error of six biological replicates (^∗^*P* < 0.05; ^∗∗^*P* < 0.01).

Representative FM plants were selected form every replicate of CK and LN treatments. Roots were separated from shoots after harvest to analyze whole-root acclimation to the LN stress. Scanned roots showed high degree of fibrousness (**Figures 5A,B**), with a network of crown roots and lateral roots. The root response to LN was very prominent, as indicated by significantly less crown and lateral roots (**Figures 5C,D**). There was a significant decrement in lateral root density, crown root length, lateral root length, and total root length under LN (**Figures 5E–H**). However, average root diameter of LN plants was significantly greater compared to that of control plants (**Figure 5I**).

**FIGURE 6:**
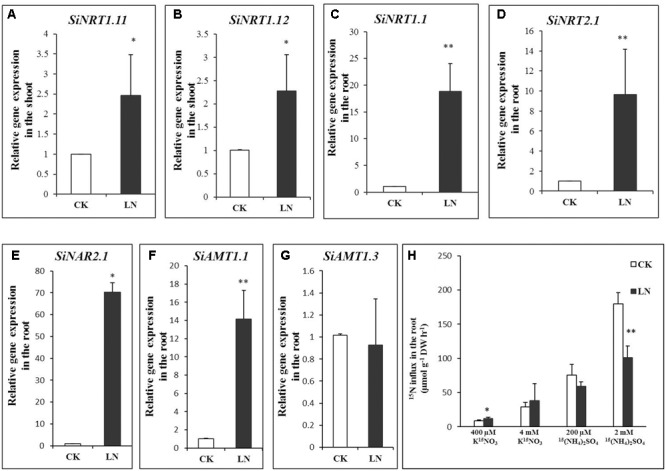
Gene expression and nitrogen (N) uptake in FM. Relative expression of SiNRT1.11 **(A)** and SiNRT1.12 **(B)** in the shoot and that of SiNRT1.1 **(C)**, SiNRT2.1 **(D)**, SiNAR2.1 **(E)**, SiAMT1.1 **(F)**, and SiAMT1.3 **(G)** in the root under CK and LN. ${\mathrm{NH}}_{4}^{+}$ and ${\mathrm{NO}}_{3}^{–}$ uptake **(H)** by CK and LN plants when exposed to contrasting levels of ^15^(NH_4_)_2_SO_4_ or K^15^NO_3_ for 10 min. Error bars represented standard error of four biological replicates for gene expression and eight replicates for N uptake (^∗^*P* < 0.05; ^∗∗^*P* < 0.01).

Plants enhance N uptake by up-regulating expression of NRT1.1 (Okamoto et al., 2003), NRT2.1 (Cerezo et al., 2001; Filleur et al., 2001), and NAR2.1 (Laugier et al., 2012; Pii et al., 2016) for root uptake under N limitation while up-regulating NRT1.11 and NRT1.12 in the shoot to transfer xylem born N to phloem for N remobilization (Hsu and Tsay, 2013). To analyze whether N transport or remobilization was affected by LN, we first analyzed transcript levels of SiNRT1.1, SiNRT2.1, and NAR2.1 in the root and those of SiNRT1.11 and SiNRT1.12 (the best FM hits based on sequence homology and sequence IDs were indicated in **Table 1**) in the shoot under CK and LN conditions by qRT-PCR. As shown in **Figures 6A,B**, expression of SiNRT1.11 and SiNRT1.12 was up-regulated in the shoot of FM grown in LN. Expression levels of SiNRT1.1, SiNRT2.1, and SiNAR2.1 were dramatically up-regulated in the root grown in LN compared to CK (**Figures 6C–E**).

Ammonium uptake is mediated by a number of conserved ammonium transporters (AMTs) (Loqué and von Wirén, 2004). AMT1.1 and AMT1.3 confer 60–70% of high-affinity ammonium uptake (Yuan et al., 2007). We identified SiAMT1.1 and SiAMT1.3 according to reciprocal sequence blast and found significant up-regulation of SiAMT1.1 expression in roots under LN (**Figure 6F**), whereas the expression level of SiAMT1.3 did not change under LN compared to control (**Figure 6G**).

To analyze whether variations in gene expression contributed to potentially differential N uptake, we further analyzed transient nitrate and ammonium uptake. As expected, LN plants had enhanced ^15^NO_3_ uptake when 400 μM K^15^NO_3_ was supplied and showed similar N influx to control plants when grown in the 4 mM K^15^NO_3_ solution (**Figure 6H**). On the other hand, LN and control plants had no difference in N influx in the 200 μM ^15^(NH_4_)_2_SO_4_ solution although lower uptake was observed in LN plants when 2 mM ^15^(NH_4_)_2_SO_4_ was supplied (**Figure 6H**).

## Discussion

Nitrogen is an essential macronutrient for plant growth, development, and production (Hirel et al., 2007; Wang Y.Y. et al., 2012). As a consequence of natural or anthropogenic factors, either inorganic or organic N is distributed in the soil in a highly heterogeneous or patchy manner (Hodge, 2004; Wang et al., 2007). Plants sense external N availability and respond accordingly via hierarchical morphological, physiological, and molecular adaptations. Legumes enhance N capture by nodulation and N fixation (Postgate, 1998), while many non-legume crops, e.g., maize, enhance axial and lateral root growth for N forage or uptake when grown in local low-N environment for a short period (Wang et al., 2003; Chun et al., 2005a) although long-term low N eventually inhibits both shoot and root growth (Chun et al., 2005b; Guo et al., 2005; Goron et al., 2015). Rice and wheat also have longer roots in response to N starvation (Cai et al., 2012; Guo et al., 2014). However, different adaptive strategies may have arisen even in closely related species in the grass family over evolution. FM is a generally nutrient-efficient high-value cereal crop originated in China (Oelke et al., 1990). It is particularly interesting to investigate the physiological and molecular mechanisms of how FM responds to low N. Lower SPAD values (**Figure 1C**) and N concentrations (**Figures 2A,B**) indicated that FM plants were under the N stress.

### A Smaller Root System Was in Contrast to Enhanced Biomass Accumulation under Low N

Maize seedlings have larger root system as an obvious morphological response to LN (Han et al., 2015). However, the LN FM seedling had a smaller root system (**Figure 1B**). The specific root length of a 3-week FM seedling grown in the full nutrient solution was 90198 cm g^-1^ of root DW (over 30 times of that of maize hybrid ZD958 and inbred Z58 and Chang7-2 seedlings; Han et al., 2015) while for LN seedlings it was 46852 cm g^-1^ of root DW (Supplementary Table 2) (over 10 times of that of seedlings of above three maize lines; Han et al., 2015). It is probably unfavorable or much more costly for such a large root plant to further enlarge its absorption surface when subjected to nutrient stresses. Alternatively, the total root length of FM seedlings decreased from 3152 to 2771 cm under LN on average (**Figure 5H** and Supplementary Table 2). Such decrease was directly caused by co-reduction in crown root length and lateral root length as a result of decreases in the number of crown and lateral roots and the lateral root density (**Figures 5C–G**). In contrast to significant decreases in root length, the root DW significantly increased (**Figure 1D**), together with the significant increases in the root/shoot ratio (**Figure 1F**), indicating systemically more carbon allocation toward the root in FM under LN for the sake of compensatory N uptake at the cost of reduced shoot growth. Enhanced carbon allocation toward the root was rather for root thickening than for root enlargement, as indicated by significant increases in average diameter of the root under LN (**Figure 5I**). Thicker roots may have wider xylem vessels or other favorable anatomical alterations facilitating nutrient transport. Further studies are required to investigate the underlying physiological and molecular mechanisms, and it remains unclear how FM roots respond to the long-term LN stress. Interestingly, if only nitrate-N (without ammonium) was supplied, LN causes reduction in total root length along with the number of crown and lateral roots in maize seedlings while increases the crown and lateral root length (Wang et al., 2004; Tian et al., 2008; Gao et al., 2015), indicating that plants respond to LN very differently depending on species, developmental stages, and the N form. In particular, FM has a fibrous root system which is completely different from that of maize and may have developed unique LN adaptive features over the evolutionary history. Lastly, reduction in crown root growth in FM under LN was similar to suppression of crown root growth in *Setaria viridis*, a domesticated relative of *S. italica*, under water deficiency (Sebastian et al., 2016).

### Root Responses to LN Were Coupled with Complicated Variations in Hormone Accumulation

Hormones are crucial regulators of plant development, growth, and adaptation to environmental cues (Wolters and Jürgens, 2009). Little is known about how hormones are involved in FM responses to N deficiency. Auxin regulates cell division, elongation, and differentiation during plant development and growth (Reed, 2001), as well as shoot to root N signaling (Forde, 2002). Blockage of auxin biosynthesis or signaling frequently causes severe developmental defects or growth retardation as stated “no growth without auxin” by Went and Thimann (1937). In maize, N limitation reduces auxin accumulation in the ear and represses ear growth (Yu et al., 2016). The auxin concentration in phloem sap of maize tissues is also closely related to nitrate supply (Tian et al., 2008). Auxin may function to repress longitudinal growth and promote lateral root proliferation to reshape root architecture (Herrera et al., 2015). In our study, the auxin concentration was significantly lower in the root and shoot under LN (**Figures 4A,E**). We reasoned that the smaller root system under N limitation may be partially due to reductions in auxin accumulation. Cytokinins regulate N signaling (Ashikari et al., 2005; Sakakibara, 2006), and biosynthesis and maintenance of auxin (Jones et al., 2010). Significant decreases in cytokinins accumulation in the root were well in agreement with the smaller root system of the LN plants (**Figures 4B,F**). Notably, the local response (cytokinin decreases in the root) did not override the systemic response of FM to N limitation (more carbon allocation toward the root). GAs are important for organ differentiation (Yamaguchi, 2008). The GA concentration was higher under LN in the root and shoot (**Figures 4C,G**). High nitrate treatment repressed GA3 levels while the LN treatment enhanced GA3 levels in *Arabidopsis thaliana* (Liu et al., 2013). GA accumulation increases in the maize ear 1 week before silking under LN (Yu et al., 2016). Increases in GA accumulation in the root of FM under LN may promote tissue differentiation and anatomical changes for root thickening (**Figures 4C,G**). ABA mediates adaptive responses of plants to N limitation and other environmental stresses (Chin and Beevers, 1970; Santner et al., 2009). ABA concentrations increased in the N-deficient maize ear from silking to 2 weeks after silking (Yu et al., 2016). Enhanced accumulation of ABA (**Figures 4D,H**) could be an essential hormonal regulatory mechanism of root adaptation to N limitation in FM although much more work is needed to further dissect underlying molecular mechanisms.

### Up-regulation of Nitrate Transporters Probably Favored Nitrate Uptake and Remobilization

Nitrate dominates in maize, wheat, and millet fields (Marschner, 1995), and nitrate absorption and remobilization is critical for crop development and grain production in these crop fields (Miller et al., 2007; Gojon et al., 2009; Forde and Walch-Liu, 2009; Krouk et al., 2010a). Nitrate is absorbed from soil solution into root cells by specific sets of NRT1 and NRT2 family transporters (Tsay et al., 2007; Miller et al., 2009). NRT1.1 is a nitrate sensor and dual-affinity nitrate transporter and NRT2.1 is a high-affinity nitrate transporter (Tsay et al., 2007; Miller et al., 2009). NRT2 interacts with NAR2-like proteins (NAR2.1/NRT3.1) for nitrate uptake (Okamoto et al., 2006; Orsel et al., 2006). NAR2.1 knockout plants fail to survive on the low nitrate medium (Okamoto et al., 2006; Orsel et al., 2006; Wirth et al., 2007; Yong et al., 2010). In our study, expression of SiNRT1.1 (an NRT1 family member), SiNRT2.1 (a putative high-affinity nitrate transporter), and SiNAR2.1 and high-affinity nitrate influx were up-regulated in the root of LN plants (**Figures 6C–E,H**), indicating that FM may enhance nitrate uptake by up-scaling transporter expression and functioning in the root. Similar to signaling roles of NRT1.1 and NRT2.1 (Vidal and Gutierrez, 2008; Gojon et al., 2009; Krouk et al., 2010b), up-regulation of SiNRT1.1 and SiNRT2.1 might have signaling roles in triggering other physiological and molecular responses. NRT1.11 and NRT1.12 are involved in xylem-to-phloem nitrate transfer and N redistribution in leaves (Hsu and Tsay, 2013). Up-regulation of SiNRT1.11 and SiNRT1.12 in the shoot under LN (**Figures 6A,B**) favored acceleration of N transfer and remobilization toward younger leaves/parts within the aboveground tissue as a fundamental strategy to cope with the LN stress. One possibility was that FM triggered simultaneous up-regulation of above five nitrate transporters as a systemic LN response; or we speculated that up-regulation of SiNRT1.1 or SiNRT2.1 in the root somehow coordinated up-regulation of SiNRT1.11 and SiNRT1.12 to optimize N allocation for maximal shoot growth and development as a measure to encounter LN availability.

On the other hand, only AMT1.1 expression is up-regulated under N limitation in Arabidopsis (Engineer and Kranz, 2007) although expression of both AMT1.1 and AMT1.3 is up-regulated under LN in rice (Kumar et al., 2003; Sonoda et al., 2003). Similar to that in Arabidopsis, SiAMT1.3 expression remained unchanged and SiAMT1.1 expression was significantly enhanced under LN (**Figures 6F,G**). However, we failed to observe significant increases in high-affinity ammonium influx (**Figure 6H**) probably due to post-translational (Yuan et al., 2013) or negative feedback regulation of SiAMT1.1. Lastly, high levels of ammonium (i.e., 2 mM) are unusual in the FM field, and low-affinity ammonium transport and corresponding lower ammonium uptake by LN plants (**Figure 6H**) remained to be further investigated in the future.

### More Soluble Proteins Accumulated in the N-Deficient Root

Amino acids and proteins are important compounds during N metabolism and storage (Miller and Cramer, 2004; Canãs et al., 2009). As expected, the concentration of free amino acids and soluble proteins decreased in the shoot as a result of N limitation whereas in the root, we observed a significant increase in soluble protein accumulation (**Figures 3A–D**), similar to our previous results in the maize ear (Yu et al., 2016). One possibility was that such proteins were co-transported to the root with enhanced carbohydrate allocation. Alternatively, N utilization efficiency (the cumulative biomass production per unit of N, g DW g^-1^ N; Han et al., 2015) and the C/N ratio increased in the root under N limitation (**Figures 2D,F**). “Excessive” soluble proteins in the root may be a N source for the shoot to ensure N demand of photosynthetic apparatuses and processes. Thus, FM may allocate more N toward the shoot for photosynthesis and carbon fixation and more carbon toward the root facilitating nutrient uptake and translocation. Related enzyme and transporter activities and gene expression patterns in FM under LN will further elaborate this hypothesis in future studies. The imbalance between carbon fixation and N assimilation leads to carbohydrate accumulation (Masclaux-Daubresse et al., 2005). Sugars accumulate in plant tissues as a consequence of N deficiency (Tercé-Laforgue et al., 2004). Significantly more soluble sugars accumulated in the shoot and root of LN plants (**Figures 3E,F**) probably as a systemic response.

## Conclusion

Unlike its cereal relatives, FM reduced total root length although it enhanced biomass accumulation in the root under N limitation. LN plants had higher C/N and R/S ratios, NUtE, soluble protein, and soluble sugar concentrations in spite of a shorter root system. Up-regulation of SiNRT1.1, SiNRT2.1, and SiNAR2.1 expression and nitrate influx in the root and that of SiNRT1.11 and SiNRT1.12 expression in the shoot manifested a putative mechanistic pathway by which FM adapted to LN conditions: enhancing nitrate uptake by thickened roots as well as nitrate translocation in the shoot to maximize shoot functioning.

## Author Contributions

XL, FZ, XD, and FN designed the research. FN, ZA, RW, JH, QS, and FC performed the research. XL and FN analyzed the data. FN and XL wrote the paper. XD and FZ revised the manuscript. All authors approved the final manuscript.

## Conflict of Interest Statement

The authors declare that the research was conducted in the absence of any commercial or financial relationships that could be construed as a potential conflict of interest.
