# Supplementary material for: Foxtail Millet [Setaria italica (L.) Beauv.] Grown under Low Nitrogen Shows a Smaller Root System, Enhanced Biomass Accumulation, and Nitrate Transporter Expression
Source: Front Plant Sci. 2018 Feb 22;9:205. doi: 10.3389/fpls.2018.00205 (PMC5826958; doi:10.3389/fpls.2018.00205)
Supplement: Supplementary file 1 [file Table_1.DOC]

| **Supplementary Table 1|** **Percentage changes in the N concentration and NUtE in the shoot and root** | | | | | | | | |
| --- | --- | --- | --- | --- | --- | --- | --- | --- |
| **Treatment** | **Shoot N concentration**  **(g kg^-1^)** | **Percentage**  **Change**  **(%)** | **Root N concentration**  **(g kg^-1^)** | **Percentage**  **Change**  **(%)** | **shoot NUtE**  **(g g^-1^)** | **Percentage**  **Change**  **(%)** | **root NUtE**  **(g g^-1^)** | **Percentage**  **Change**  **(%)** |
| **CK** | 40.28 ± 3.09a | -69 | 41.08 ± 7.04a | -54 | 25.51 ± 1.77b | 216 | 27.48 ± 3.91b | 98 |
| **LN** | 12.53 ± 2.41b |  | 18.99 ± 1.48b |  | 80.65 ± 3.58a |  | 54.48 ± 4.70a |  |
| Different letters after the values within the same column indicated significant differences (P < 0.05). Percentage change = [(value under LN – Value under CK)/Value under CK] * 100%. | | | | | | | | |
