# Supplementary material for: Foxtail Millet [Setaria italica (L.) Beauv.] Grown under Low Nitrogen Shows a Smaller Root System, Enhanced Biomass Accumulation, and Nitrate Transporter Expression
Source: Front Plant Sci. 2018 Feb 22;9:205. doi: 10.3389/fpls.2018.00205 (PMC5826958; doi:10.3389/fpls.2018.00205)
Supplement: Supplementary file 2 [file Table_2.DOC]

| **Supplementary Table 2| Percentage changes in total root length and specific root length** | | | | |
| --- | --- | --- | --- | --- |
| **Treatment** | **Total root length (cm)** | **Percentage change (%)** | **Specific root length**  **(cm g^-1^ root DW)** | **Percentage change (%)** |
| **CK** | 3152 ± 192a | -12 | 90198 ± 15310a | -48 |
| **LN** | 2771 ± 80b |  | 46852 ± 6741b |  |
| Different letters after the values within the same column indicated significant differences (P < 0.05). Percentage change = [(value under LN – Value under CK)/Value under CK] * 100%. | | | | |
