# Supplementary material for: Foxtail Millet [Setaria italica (L.) Beauv.] Grown under Low Nitrogen Shows a Smaller Root System, Enhanced Biomass Accumulation, and Nitrate Transporter Expression
Source: Front Plant Sci. 2018 Feb 22;9:205. doi: 10.3389/fpls.2018.00205 (PMC5826958; doi:10.3389/fpls.2018.00205)
Supplement: Supplementary file 3 [file Table_3.DOC]

| **Supplementary Table 3| Percentage changes in the concentration of free amino acids in the shoot and root** | | | | |
| --- | --- | --- | --- | --- |
| **Treatment** | **Free amino acids (Shoot)**  **(µg g^-1^ FW)** | **Percentage change (%)** | **Free amino acids (Root)**  **(µg g^-1^ FW)** | **Percentage change (%)** |
| **CK** | 357.45 ± 42.94a | -67 | 384.42 ± 51.91a | -73 |
| **LN** | 118.89 ± 10.56b |  | 103.39 ± 4.49b |  |
| Different letters after the values within the same column indicated significant differences (P < 0.05). Percentage change = [(value under LN – Value under CK)/Value under CK] * 100%. | | | | |
