# Supplementary material for: Foxtail Millet [Setaria italica (L.) Beauv.] Grown under Low Nitrogen Shows a Smaller Root System, Enhanced Biomass Accumulation, and Nitrate Transporter Expression
Source: Front Plant Sci. 2018 Feb 22;9:205. doi: 10.3389/fpls.2018.00205 (PMC5826958; doi:10.3389/fpls.2018.00205)
Supplement: Supplementary file 4 [file Table_4.DOC]

| **Supplementary Table 4| Percentage changes in concentrations of soluble proteins in the shoot and root** | | | | |
| --- | --- | --- | --- | --- |
| **Treatment** | **Soluble proteins (shoot)**  **(mg g^-1^ FW)** | **Percentage change (%)** | **Soluble proteins (Root)**  **(mg g^-1^ FW)** | **Percentage change (%)** |
| **CK** | 5.57 ± 0.18a | -49.91 | 1.89 ± 0.27a | 57.14 |
| **LN** | 2.79 ± 0.33b |  | 2.97 ± 0.25b |  |
| Different letters after the values within the same column indicated significant differences (P < 0.05). Percentage change = [(value under LN – Value under CK)/Value under CK] * 100. | | | | |
