# Supplementary material for: Foxtail Millet [Setaria italica (L.) Beauv.] Grown under Low Nitrogen Shows a Smaller Root System, Enhanced Biomass Accumulation, and Nitrate Transporter Expression
Source: Front Plant Sci. 2018 Feb 22;9:205. doi: 10.3389/fpls.2018.00205 (PMC5826958; doi:10.3389/fpls.2018.00205)
Supplement: Supplementary file 5 [file Table_5.DOC]

| **Supplementary Table 5| Percentage changes in the concentration of total soluble sugars in the shoot and root** | | | | |
| --- | --- | --- | --- | --- |
| **Treatment** | **Total Soluble Sugars (shoot)**  **(mg g^-1^ FW)** | **Percentage change (%)** | **Total Soluble Sugars (Root)**  **(mg g^-1^ FW)** | **Percentage change (%)** |
| **CK** | 4.59 ± 1.36b | 64 | 0.28 ± 0.07b | 32 |
| **LN** | 7.53 ± 0.31a |  | 0.37 ± 0.04a |  |
| Different letters after the values within the same column indicated significant differences (P < 0.05). Percentage change = [(value under LN – Value under CK)/Value under CK] * 100. | | | | |
